# Establishment of an infectious clone of the porcine transmissible gastroenteritis virus and a study on the location and function of accessory protein 3

**DOI:** 10.3389/fcimb.2025.1609022

**Published:** 2025-06-11

**Authors:** Zhenye Hao, Lifei Liu, Shuai Wang, Yanping Jiang, Jiaxuan Li, Wen Cui, Li Wang, Yijing Li

**Affiliations:** ^1^ College of Veterinary Medicine, Northeast Agricultural University, Harbin, China; ^2^ Heilongjiang Key Laboratory for Animal Disease Control and Pharmaceutical Development, Harbin, China

**Keywords:** TGEV, ORF3, reverse genetics, viral replication, immunogenicity

## Abstract

**Introduction:**

Transmissible gastroenteritis virus (TGEV), as the causative agent of TGE, causes huge economic losses in the pig industry worldwide.

**Methods:**

In this study, we successfully constructed a reverse genetic system for the TGEV TH-98 strain and rescued the recombinant virus rTH-98-Δ3-COE-HA based on this system.

**Results:**

The results showed that ORF3 is a non-structural protein and does not exist in mature virions. Therefore, rTH-98-Δ3-COE-HA induced inflammatory cytokine expression in IPEC-J2 cells, but the expression levels were significantly lower than those triggered by TH-98 and rTH-98, indicating that TGEV ORF3 may play a critical role in inducing the host immune response. To detect whether the exogenous protein expressed in the rTH-98-Δ3-COE-HA has immunogenicity, the results showed that the levels of antibodies were significantly increased in mice. rTH-98-Δ3-COE-HA can induce a specific immune response in the host body against its expressed exogenous proteins.

**Discussion and conclusion:**

Our study provides important clues for revealing the role of ORF3 in virus replication and pathogenesis, laying a theoretical and material foundation for the construction of recombinant viral multi-valent vaccines.

## 1 Introduction

Porcine transmissible gastroenteritis virus (TGEV), a contagious porcine enteropathogenic virus, belongs to the family of alpha coronaviruses within the Nidovirales order (Wang et al., 2018; Qian et al., 2019; Dong et al., 2020). TGEV can cause severe intestinal infectious diseases in piglets under 7 days of age, with vomiting, diarrhea, and dehydration as the main symptoms (Enjuanes et al., 2005; Becares et al., 2014). Porcine-transmissible gastroenteritis still exists in intensive pig farms, posing a severe threat to the pig industry (Anand et al., 2003; Lv et al., 2014). Currently, TGEV is one of the most important diseases threatening pork production (Yang et al., 2018). Therefore, it is urgent to establish a safe coronavirus reverse genetics system.

Apart from coding for structural proteins and non-structural proteins involved in virus genome transcription and replication, coronaviruses also encode a series of unique accessory proteins (Wang et al., 2018; Rangan et al., 2020; Juanes-Velasco et al., 2022). In 2003, Sabel et al. reported that recombinant viruses with specific fluorescence were rescued after replacing TGEV open reading frame (ORF3) with green fluorescent protein (GFP) through the reverse genetic operating system (Sola et al., 2003). In addition, research has shown that ORF3 is involved in regulating the host’s innate immune response, but its role in virus replication and pathogenesis is not yet clear. Although it is widely believed that accessory proteins are non-essential for virus replication *in vitro*, they play a significant role in the specific interactions between the virus and host (Clementz et al., 2010; Malik, 2020). Therefore, fully exploring the function of accessory proteins will help us understand coronavirus pathogenesis and develop effective antiviral drugs and vaccines.

Reverse genetics has provided a novel platform to manipulate viral genomes (Cheng et al., 2016; Zhang et al., 2019). Reverse genetics is a molecular biology method for studying viral protein function, identifying virulence factors, developing new vaccines, and screening antiviral drugs to explore these genotype-phenotype associations (Johnson et al., 2018; Chey et al., 2021). Ribes et al. replaced the S gene of TGEV with the S gene of MHV and mice immunized with the recombinant virus produced incomplete protection against rotavirus infection, indicating the potential of coronaviruses as an immune vector (Ribes et al., 2011). Herein, we successfully constructed a reverse genetic operation system for the TGEV TH-98 strain and rescued the recombinant virus rTH-98-Δ3-COE-HA based on this system. In addition, ORF3 is not necessary for TGEV replication, but it promotes TGEV replication. Our research provides an experimental platform for the study of the structure and function of coronaviruses.

## 2 Materials and methods

### 2.1 Cells and viruses

ST cells were cultured in Dulbecco’s modified Eagle’s medium (DMEM; Gibco, 12491015, USA) supplemented with 10% fetal bovine serum at 37°C with 5% CO_2_. TGEV strain TH-98 (GenBank accession number KU729220) was propagated at 37°C in a 5% CO_2_ incubator in DMEM supplemented with 2% fetal bovine serum (Gibco, 10099141, USA).

### 2.2 Amplification of TGEV cDNAs and sequence analysis

Total RNA was reverse transcribed by reverse transcriptase and random primers (Takara, 639574, Japan). All the fragments were amplified by polymerase chain reaction (PCR) with Phanta Super Fidelity DNA polymerase (Vazyme, P510, China). Several site mutations, including T6228C, T7349C, T8823A, and A23828G, were found and preserved in the relevant clone. T1514G was mutated by overlapping PCRs to insert the *Mlu* I site and distinguish the parental TGEV. As per a previous study, TGEV ORF3 was replaced with the PEDV COE gene (Wang et al., 2019) instead of the original sequence (24800 to 25666).

### 2.3 TGEV subclones

The TGEV genome was divided into six successive fragments (A to F), and the fragments were amplified by RT-PCR with specific primers. Fragments A to F were cloned into the pBlunt-Simple vector (Transgen, Beijing, China). In particular, the CMV promoter gene and fragment A were cloned to maintain an *Fse* I site. Fragment B was cloned with the *Mlu* I site and *Pme* I site. Fragment C was cloned with the *Pme* I site and *Bsu36* I site. Fragment D was cloned with the *Bsu36* I site and *Avr* II site. Fragment E was cloned with the *Avr* II sites. Fragment F was cloned with the *Avr* II site and *Asc* I site introduced at the 3’ terminal of the TGEV genome. Subclone F also carried the synthesized necessary sequences (Wang et al., 2019). The BAC plasmid was used as the final vector (kindly provided by Prof. Han Jun from the College of Veterinary Medicine and the State Key Laboratory of Agrobiotechnology, China Agricultural University).

### 2.4 Assembly of TGEV genome in pBAC

Subclone A was first digested with *Fse* I and *Mlu* I. Subclone F was then digested with *Avr* II and *Asc* I. Next, all the objective fragments were purified by gel extraction, and ligated in the order of A-F-D-E-B-C. The positive clone was designated pBAC-TGEV-Full after sequencing (Seven, Harbin, China). The E fragment with the PEDV COE gene was ligated with *Avr* II-digested pBAC-TGEV-Full. The assembly of pBAC-TGEV-Δ3-COE-HA was the same as above. Then, the relevant plasmid was transfected into ST cells to rescue the virus.

### 2.5 Indirect immunofluorescence assay and viral plaque assay

The ST cells were infected with passage 1 viruses of rTH-98 or rTH-98-Δ3-COE-HA for 24 h. The cells were fixed with -20°C cold ethanol. After being blocked, the cells were treated with TGEV N protein antibody or rabbit polyclonal antibody to HA protein (Invitrogen, USA). Then, the cells were reacted with relevant secondary antibodies. After three washes, the samples were observed using a Nikon inverted fluorescence microscope.

ST cells were infected with wild-type TGEV or rescued TGEV at different dilutions and overlaid with DMEM containing 0.5% methylcellulose. At 36 h post-infection, the medium was removed and the plaques were stained with 0.01% neutral red.

### 2.6 Electron microscopy analysis

The rescued virus cell cultures were centrifuged at 3,000 × g for 30 min to obtain the supernatant. Subsequently, the supernatant was recentrifuged at 15,000 × g for 30 min to gain virus particles and negatively stained with 2% phosphotungstic acid (pH 7.0). In addition, sucrose gradient centrifugation-purified rTH-98-Δ3-COE-HA supernatant was incubated with rabbit polyclonal antibody to HA protein (Invitrogen, Carlsbad, CA, USA). Finally, the samples were examined by transmission electron microscopy (Hitachi, Tokyo, Japan).

### 2.7 Animal experiments

To evaluate the immunogenicity of the rescued viruses (rTH-98 and rTH-98-Δ3-COE-HA) and clarify the localization of ORF3, 35-day-old female BALB/c mice (n = 60) were provided with adequate water and food for the standard. The temperature and humidity of the breeding house was kept consistent (temperature: 23 ± 1°C; humidity: 50–60%) during the experiments. All efforts were made to minimize suffering and euthanasia was performed. All the experimental procedures and animal management procedures were approved by the Institutional Committee of the Northeast Agricultural University for the Animal Experiments (SRM-11). Mice were immunized with 200 µL PBS, rTH-98-Δ3-COE-HA (1×10^7^ TCID_50_/mL), rTH-98 (1×10^7^ TCID_50_/mL), or 200 µg purified rTH-98-Δ3-COE-HA (10 mice per group). Each mouse was immunized twice and there was a 14-day interval between the two immunizations.

### 2.8 Enzyme-linked immunosorbent assay and neutralizing antibody titers in serum

Changes in IgG in serum were detected by indirect enzyme-linked immunosorbent assay (ELISA). Briefly, after overnight storage at 4°C, PEDV COE protein or TGEV N protein was coated on an ELISA plate. After being blocked, samples were incubated for 2 h at 37°C. Subsequently, the secondary antibodies (1:5000) (GoldenGates, 00152, China) were added and the samples were detected at 490 nm absorbance.

To determine the neutralizing titers of anti-TGEV IgG and anti-PEDV IgG in serum, serum from each immunized mouse was collected at 42 days post immunization and continuously diluted. The mixture of diluted serum and 50 µL of TCID_50_ TGEV and PEDV was incubated and then transferred to the corresponding cell monolayer. The formation of specific CPE was observed after 48 h.

### 2.9 Statistical analysis

Statistical analyses were performed using a one-way analysis of variance (ANOVA) test in GraphPad Prism 8 software (GraphPad Software, Inc.). Results were expressed as mean ± SD. A *P*-value of less than 0.05 indicated that the difference was statistically significant.

## 3 Results

### 3.1 Construction of the infectious cDNA clones of TH-98

**Figure 1:**
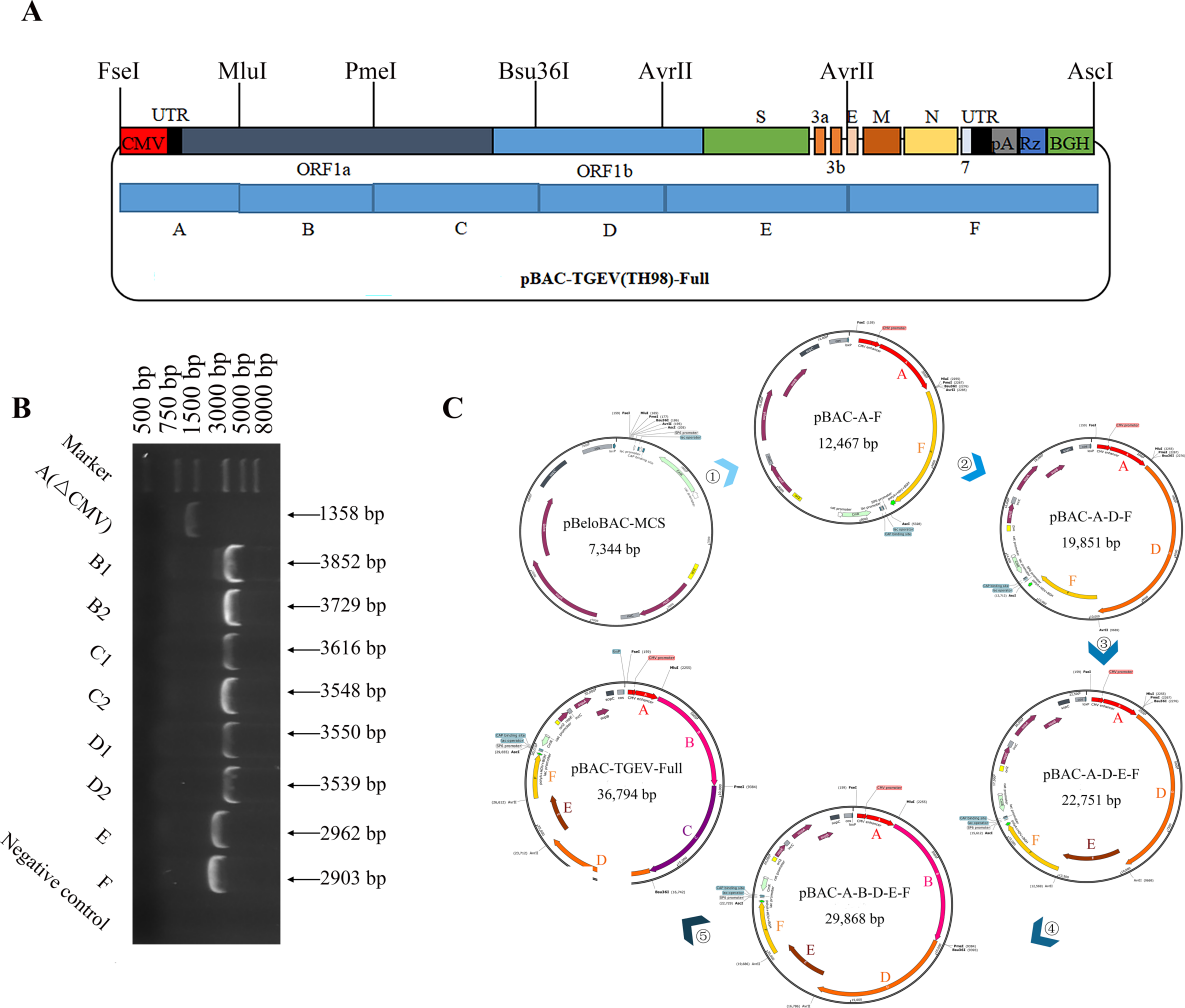
Construction of the infectious cDNA clones of TH-98. **(A)** The cDNA segments of TGEV TH-98 were cloned into the BAC plasmid to assemble the full-length cDNA clones. **(B)** The TH-98 genome was divided into six segments (A, B, C, D, E, and F) and ligated into a pBlunt-simple vector. Electrophoresis identification of the pBlunt-simple vectors was conducted. **(C)** Construction order of the TGEV reverse genetic system.

The BAC plasmid (pBeloBAC11) was used to assemble the full-length cDNA clones of TH-98. In this system, the TGEV genome is controlled by the CMV promoter to synthesize mRNA. Moreover, HDVRz and BGH sequences were added to the 3’ end of the TGEV genome to assist in the precise processing of the poly (A) tail (Li et al., 2017). For ease of cloning, the TH-98 genome was divided into six fragments (A-F), with their cleavage sites and relative enzymes shown in **Figure 1A**. Notably, the CMV promoter sequence was fused to the 5’ end of the genome and the PolyA tails(28A)-HdvRz-BGH sequence was fused to the 3’ end of the genome. All the PCR amplified fragments were ligated into the pBlunt-simple vector and subsequently examined by DNA sequencing (**Figure 1B**). T1514G(genetic marker)in fragment A was synonymously mutated to distinguish it from the parental TGEV. Subsequently, six fragments were assembled into pBAC-MCS by homologous recombination in the order shown to synthesize pBAC-TGEV(TH-98)-Full (**Figure 1C**).

### 3.2 Recovery and identification of the rescued virus

**Figure 2:**
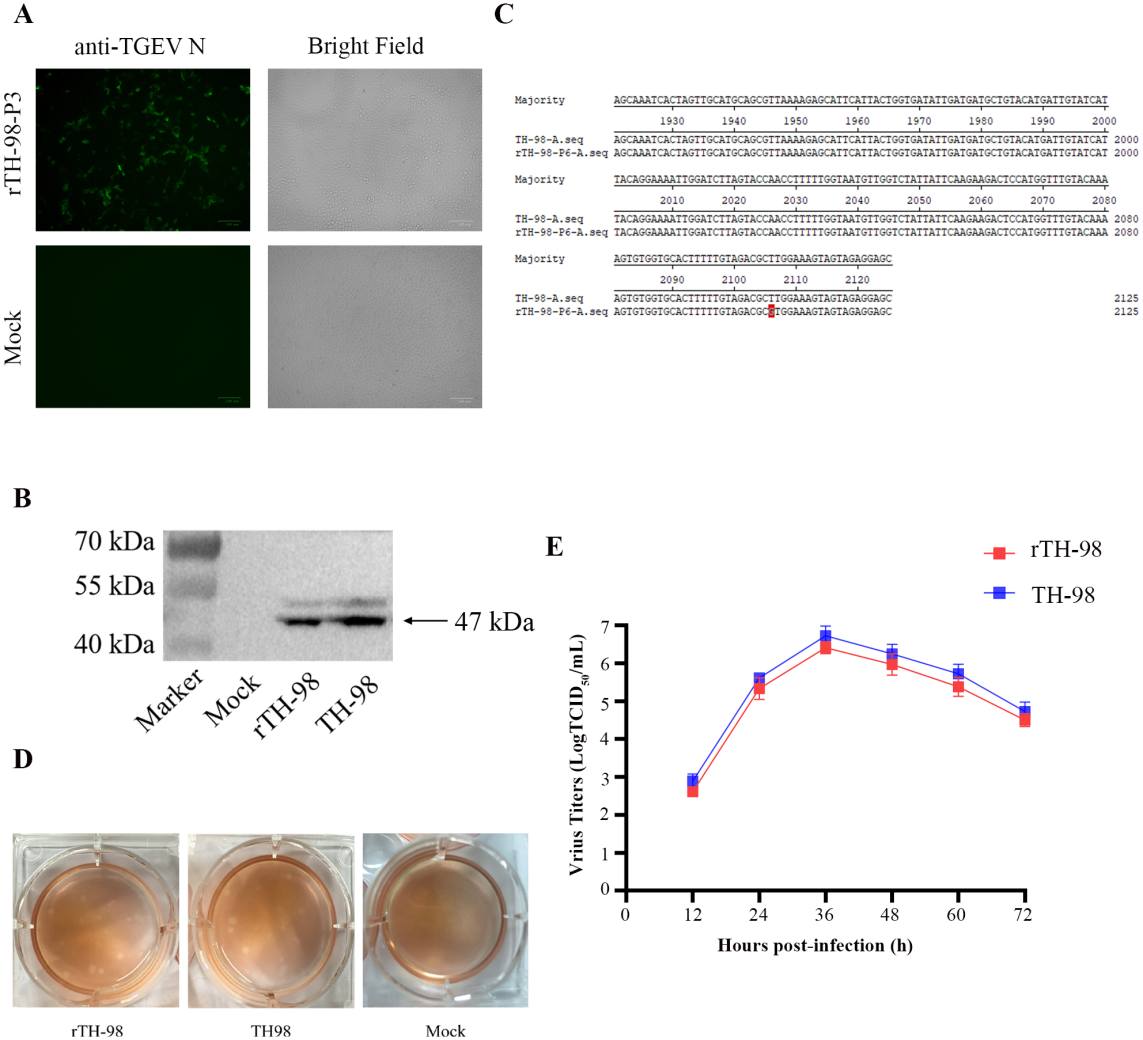
Recovery and identification of the rescued virus. **(A)** Indirect immunofluorescence staining of the rescued virus with mouse monoclonal antibody to TGEV N protein. **(B)** The expression of the TGEV N protein in ST cells infected with the rescued virus, WT TGEV, or mock-infected was analyzed by Western blotting using mouse monoclonal antibody to TGEV N protein. **(C)** Detection of the genetic marker by sequencing of the rescued virus. **(D)** Comparison of plaque sizes and morphology of WT TGEV and the infectious clone-derived viruses on ST cells at 72 h post-infection. **(E)** Growth curve of rTH-98 and TH-98 in ST cells (MOI=0.01).

We transfected the plasmid pBAC-TH-98 into ST cells. After 48–96 h, the CPE was similar to its parental virus TH-98 (**Figure 2A**). The corresponding virus was subsequently named rTH-98. rTH98 was identified by the indirect immunofluorescence assay (IFA) (**Figure 2A**) and Western blot assay (**Figure 2B**) and by sequencing the relevant genetic marker fragments (**Figure 2C**). Therefore, we succeeded in rescuing TGEVs using the DNA launch system.

Next, we evaluated the replication properties of the rescued virus. The plaque assay results showed that rTH-98 had a similar size and plaque morphology to the parental virus (**Figure 2D**). Next, we investigated the growth properties of rTH-98 using a multi-step growth assay. As shown in **Figure 2E**, the growth kinetics of rTH-98 were similar to those of the parental virus. However, at each time point, the rTH-98’s titer was slightly lower than the parental virus titer. Due to the continuous passage of the parent virus in the cells, it has adapted to the cells and the parental virus exists as quasi-species while the infectious clone-derived virus represents a relatively homogeneous population.

### 3.3 Construction of the infectious cDNA clones of TH98-Δ3-COE-HA and a rescue recombinant virus

**Figure 3:**
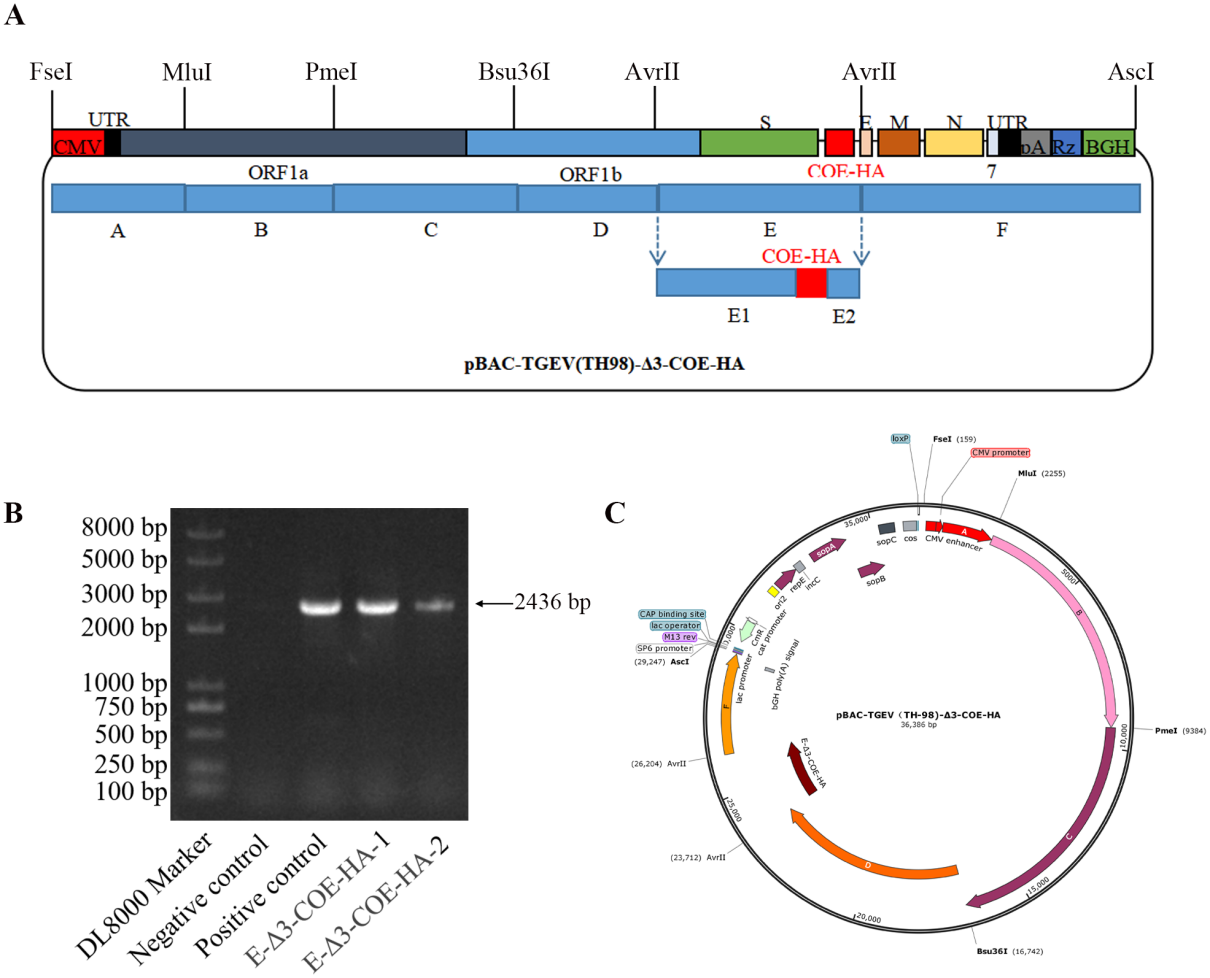
Construction of the infectious cDNA clones of TH98-Δ3-COE-HA and the rescued recombinant virus. **(A)** Model for the construction of the TH98-Δ3-COE-HA infectious clones. **(B)** Electrophoresis detection of pBAC-TH98-Δ3-COE-HA by bacterial PCR. **(C)** Schematic map of pBAC-TH98-Δ3-COE-HA.

In order to study the function of TGEV ORF3 at the viral level, a recombinant virus with ORF3 deletion was constructed using the established TGEV reverse genetic operating system. Due to the independent ORFs of ORF3a and ORF3b genes, with their TRS located near the S gene and the TRS of the E gene located in ORF3b, complete deletion of ORF3b would result in the inability of the E gene to express normally. Therefore, while retaining TRS3a and TRSE, the ORF3a and ORF3b genes in the E fragment were seamlessly cloned and replaced with the PEDV COE gene. The IS sequence was introduced at its 5’ end to enhance its transcription efficiency, and the HA tag was introduced at its 3’ end to construct the recombinant plasmid pBAC-TH-98-Δ3-COE-HA (**Figure 3A**). Through the one-step assembly of the fragments of E1-COE-HA-E2, bacterial PCR identification (**Figure 3B**), and by sequencing of the fragments carrying the COE-HA gene, a cDNA infectious clone of recombinant TGEV, named pBAC-TH98-Δ3-COE-HA, was obtained (**Figure 3C**).

### 3.4 Recovery and identification of recombinant viruses

**Figure 4:**
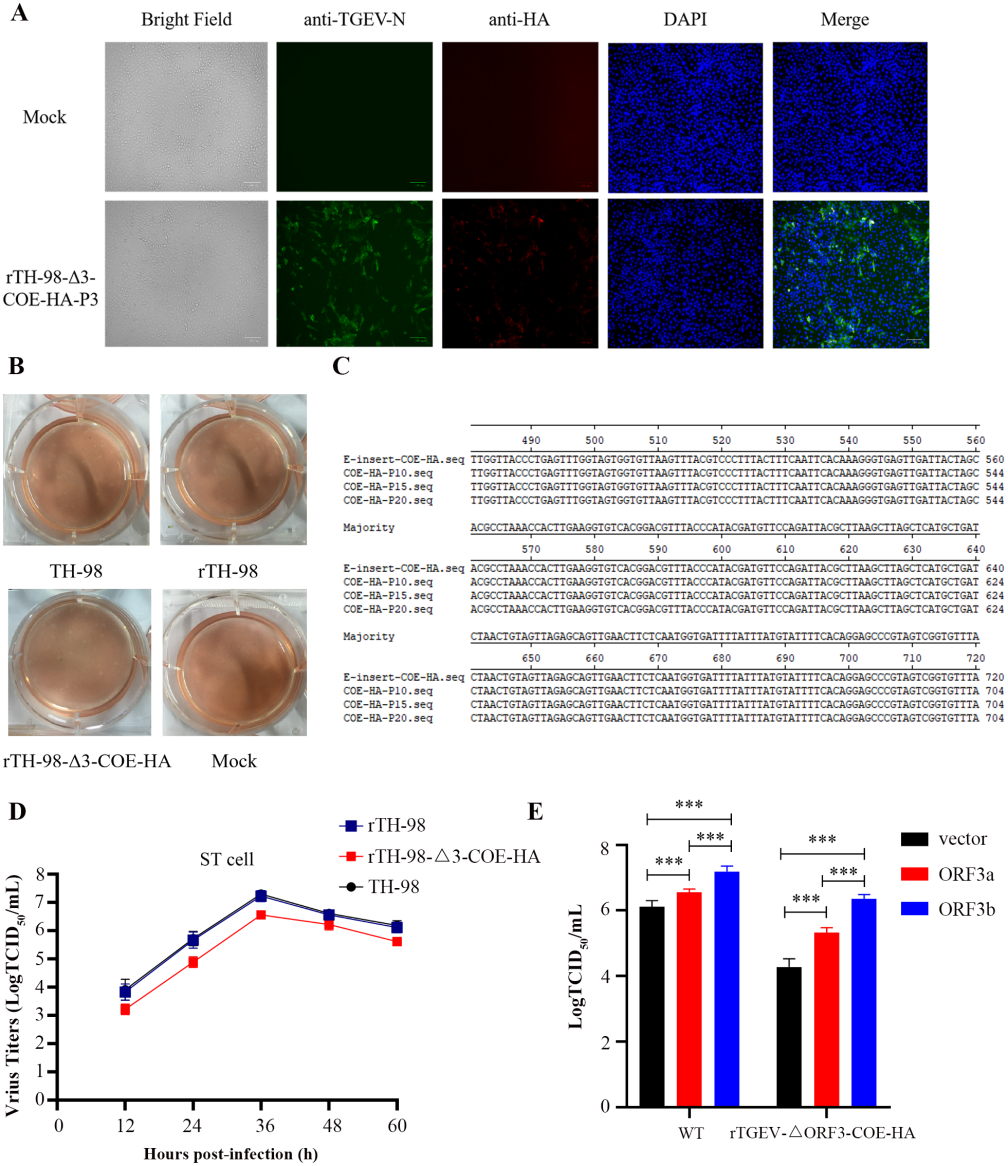
Recovery and identification of the recombinant virus. **(A)** Indirect immunofluorescence staining of rescued virus with mouse monoclonal antibody to TGEV N protein (green) and rabbit polyclonal antibody to HA protein (red). **(B)** Comparison of plaque sizes and morphology of WT TGEV and the rescued virus on ST cells at 72 h post-infection. **(C)** Identification of several passages of the recombinant virus genetic marker. **(D)** Multi-step growth curve of WT and rescued viruses on ST cells at an MOI of 0.01. **(E)** Complementation assay of rTH-98-Δ3-COE-HA infection in ST cells transiently expressing ORF3a and ORF3b. Values are the mean ± SD of three independent tests. *** represents p <0.001 between different groups.

pBAC-TH98-Δ3-COE-HA was transfected into ST cells. The CPE did not appear at passage 0. However, it appeared upon passage 3, which was similar to its parental virus, TH-98. The recombinant virus was subsequently named rTH-98-Δ3-COE-HA. rTH-98-Δ3-COE-HA was identified by the IFA (**Figure 4A**). The plaque assay showed that rTH-98 and rTH-98-Δ3-COE-HA had similar size and plaque morphology to TH-98 (**Figure 4B**). The absence of the ORF3 gene does not affect virus rescue, and ORF3 is not essential for virus replication. The RT-PCR and sequencing results showed that the recombinant viruses had not undergone mutations or deletions after 20 generations of continuous transmission (**Figure 4C**). Furthermore, rTH-98-Δ3-COE-HA had slower growth kinetics as compared to rTH-98 (**Figure 4D**). To confirm whether the decrease in recombinant rTH-98-Δ3-COE-HA titers was caused by the absence of ORF3, pCMV-HA-ORF3a, pCMV-HA-ORF3b, and empty vector pCMV-HA were transiently transfected into ST cells. Compared with ST cells transfected with empty vectors, the titers of rTH-98 and rTH-98-Δ3-COE-HA were increased in ST cells transfected with pCMV-HA-ORF3a and pCMV-HA-ORF3b, but were higher in ST cells transfected with pCMV-HA-ORF3b (**Figure 4E**). The above results indicate that neither ORF3a nor ORF3b is necessary for TGEV replication. However, ORF3a and ORF3b can affect TGEV replication *in vitro*, and ORF3b has a stronger effect than ORF3a.

### 3.5 Localization of ORF3 in TGEV

**Figure 5:**
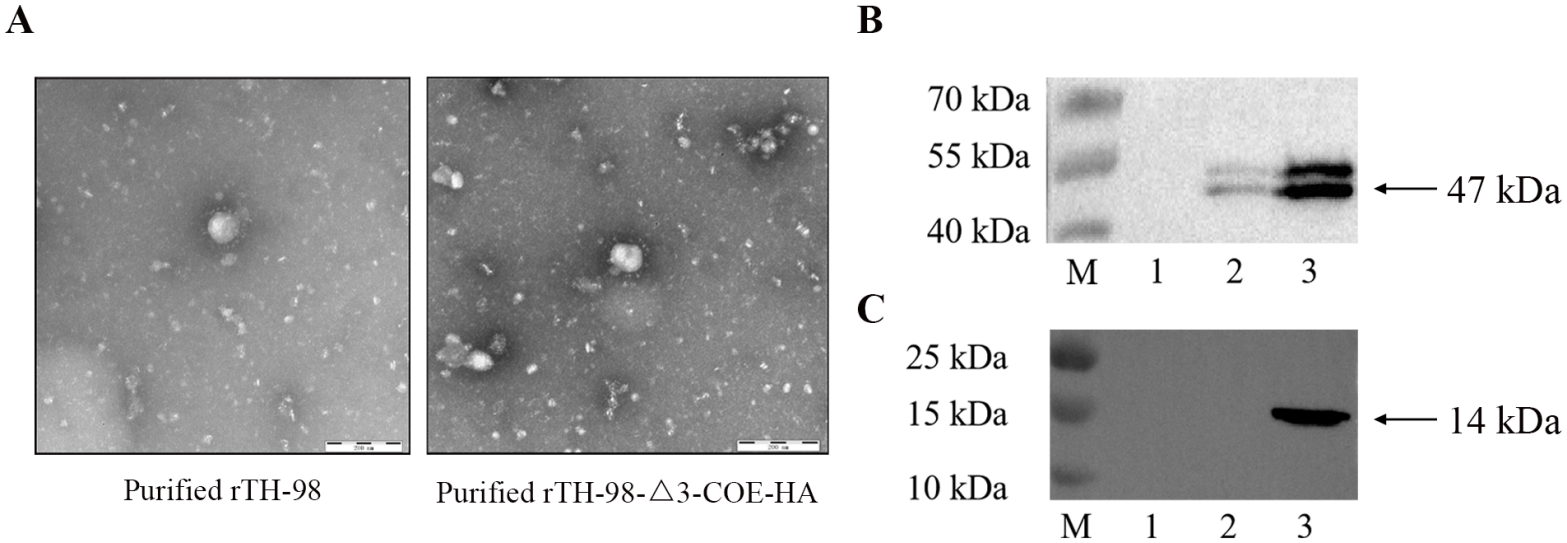
Localization of ORF3 in TGEV. **(A)** Observation of the recombinant virus by immunoelectron microscopy. **(B)** The expression of the TGEV N protein in the recombinant virus cell cultures, purified recombinant virus, or mock-infected was analyzed by Western blotting using a mouse monoclonal antibody to TGEV N protein. M, prestained protein marker; 1, mock; 2, purified recombinant virus; 3, unpurified recombinant virus. **(C)** The expression of the COE-HA protein in recombinant virus cell cultures, purified recombinant virus, or mock-infected was analyzed by Western blotting using a rabbit polyclonal antibody to the HA protein. M, prestained protein marker; 1, mock; 2, purified recombinant virus; 3, unpurified recombinant virus.

Since it is currently unknown whether ORF3 is located on mature virus particles, the recombinant virus was cultured in large quantities and purified by sucrose gradient centrifugation. HA antibodies were used for immunoelectron microscopy observation. The results showed the complete coronavirus, but no colloidal gold particles were found around the virus particles (**Figure 5A**), indicating that the COE-HA protein may not be a component of mature virus particles. Therefore, it is speculated that ORF3 is not a structural protein of TGEV. The Western blot results showed that both purified recombinant virus and infected recombinant virus cell samples showed a 47 kDa target protein band, and the size of the expressed protein was consistent with that of the N protein. However, no target size band was observed in the control group, indicating successful rescue of the recombinant virus (**Figure 5B**). The cell culture of the recombinant virus can detect a 14 kDa target protein band, and the size of the expressed protein is consistent with that of the COE-HA protein (**Figure 5C**). However, the purified recombinant virus and the control group did not show the target protein band. The above results indicate that recombinant viruses can proliferate in ST cells, and exogenous proteins can be expressed in infected cells, but they do not exist in mature virus particles.

### 3.6 Recombinant virus immunogenicity analysis

**Figure 6:**
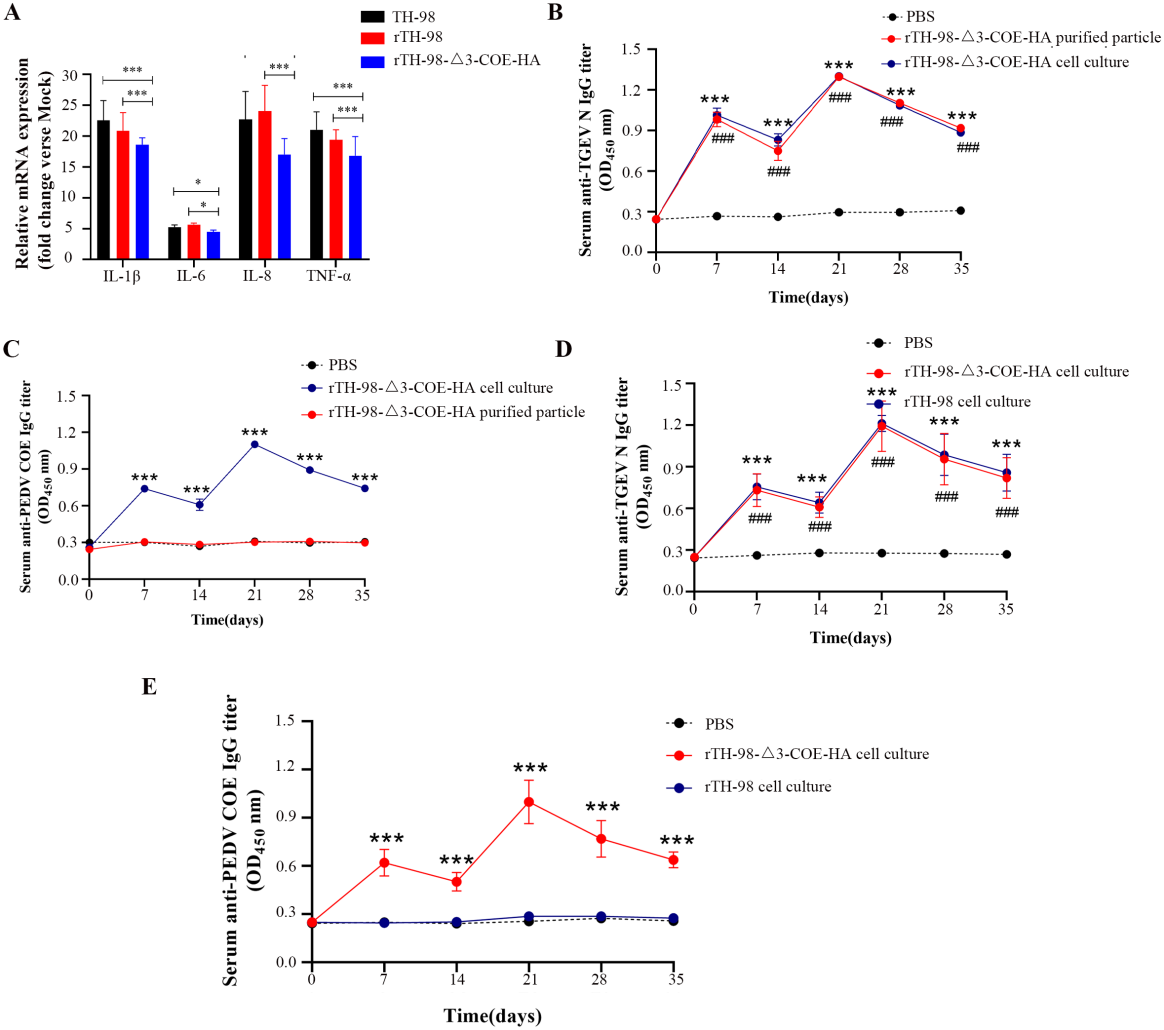
Recombinant virus immunogenicity analysis. **(A)** mRNA expression levels of IL-1β, IL-6, IL-8, and TNF-α in infected rescue virus IPEC-J2 cells. Values are the mean ± SD of three independent tests. * represents p<0.05; *** represents p <0.001 between different groups. The changes in anti-TGEV-N protein-specific IgG antibody levels in immunized mice. **(B)** Recombinant virus cell cultures and purified recombinant virus. **(C)** Recombinant virus and parental rescued virus cell cultures. The changes in anti-PEDV-COE protein-specific IgG antibody levels in immunized mice. Values are the mean ± SD of three independent tests. *** indicates a significant difference between the rTH-98-Δ3-COE-HA cell culture group and PBS group (P<0.001). ### indicates a significant difference between the rTH-98-Δ3-COE-HA purified particle group and PBS group (P<0.001). **(D)** Recombinant virus cell cultures and purified recombinant virus. **(E)** Recombinant virus and parental rescued virus cell cultures. Values are the mean ± SD of three independent tests. *** indicates a significant difference between the rTH-98-Δ3-COE-HA cell culture group and PBS group (P<0.001). ### indicates a significant difference between the rTH-98 cell culture group and PBS group (P<0.001).

To investigate whether ORF3 affects the expression of inflammatory cytokines in IPEC-J2 cells after rTH-98-Δ3-COE-HA, rTH-98, and TH-98 infection, the levels of IL-1β, IL-6, IL-8, and TNF-α were detected. The expression levels of IL-1β, IL-6, IL-8, and TNF-α in IPEC-J2 cells infected with rTH-98 and TH-98 were significantly increased (**Figure 6A**). However, the levels of inflammatory cytokines in the rTH-98-Δ3-COE-HA-infected IPEC-J2 cells were significantly lower than those in the IPEC-J2 cells infected with rTH-98 (**Figure 6A**), indicating that ORF3 may play a key role in inducing host cell immune responses.

Mice were immunized with rTH-98 cell cultures, rTH-98-Δ3-COE-HA cell cultures, purified rTH-98-Δ3-COE-HA, and PBS to detect the induction of specific IgG levels. The results showed that from 7 days post-immunization, compared with the PBS group, all the groups produced TGEV N protein-specific IgG (**Figures 6B, D**). However, only mice immunized with rTH-98-Δ3-COE-HA virus cell cultures produced specific IgG for PEDV COE protein, while mice immunized with purified virus particles did have specific IgG for PEDV COE protein (**Figures 6C, E**). To evaluate the neutralizing antibody levels produced by mice immunized with rTH-98-Δ3-COE-HA cell cultures, the neutralizing antibody levels in mice serum in the 21 days post-first immunization were detected. As shown in **Table 1**, the neutralizing antibody concentration against PEDV in mice immunized with rTH-98-Δ3-COE-HA cell cultures was 1:8. The neutralizing antibody concentration against TGEV in mice immunized with rTH-98-Δ3-COE-HA cell cultures and rTH-98 cell cultures were both 1:16. The above results showed that during the replication process of rTH-98-Δ3-COE-HA, its exogenous proteins were successfully expressed in the form of non-structural proteins and it can induce a host immune response to produce specific antibodies and neutralizing antibody. The above results indicate that TGEV ORF3 exists during the viral replication process, rather than in mature viral particles, indicating that ORF3 is a non-structural protein of TGEV and can carry and express exogenous proteins, making it an ideal insertion site.

**Table 1:** Serum neutralizing antibody titer of mice.

Group	Titer of neutralizing antibody
rTH-98-Δ3-COE-HA cell culture	1:8(PEDV)
	1:16(TGEV)
rTH-98 cell culture	<1 :2(PEDV)
	1:16(TGEV)
PBS	<1 :2(PEDV)
	<1 :2(TGEV)

## 4 Discussion

Currently, the phenomenon of co-infected TGEV and PEDV increases the difficulty of prevention and management of piglet viral diarrhea. Research has shown that the PRRSV M gene was inserted into the TGEV expression cassette, and the recombinant virus produced specific antibodies against PRRSV to resist infection with PRRSV, suggesting that a coronavirus has the potential to be a vector for expressing immunogenic genes (Becares et al., 2014). Hence, using a coronavirus as an immune vector, the introduction of foreign protein genes with the immunogenicity of other viruses could provide technical and theoretical support for the progression of multi-live virus vector vaccines.

The reverse genetic system is an efficient technology platform for life sciences to explore the relationship between genes and biological functions (Peng et al., 2018; Fang et al., 2021; Zhu et al., 2022). The coronavirus reverses the genetic system based on BAC, which has many advantages. A TGEV vector vaccine can simulate natural infection and efficiently replicate in the intestine and the TGEV genome has a strong carrying capacity. Finally, recombinant virus have strong replication ability and can activate cellular and humoral immunity (Pascual-Iglesias et al., 2019). Our study developed a TGEV reverse genetic system to rescue TGEV-PEDV COE recombinant virus, which could have the potential to become a bivalent vaccine. In our study, we used the strategy to divide the TGEV genome into six fragments, “A–F”, according to the distribution of enzyme restriction sites. Notably, the selection of enzyme restriction sites should avoid the high methylation of the site and the use of isoenzymes, improving the success rate of linearization BAC as much as possible. In the present study, a full-length infectious cDNA clone of TGEV based on BAC was assembled, and the recombinant virus was successfully rescued. When comparing the rescue virus rTH-98 and parent virus TH-98, the whole-genome sequences were identical except for the molecular markers. We observed the same pathological changes, expression of N protein, and plaque areas in ST cells. In addition, we also found similar growth dynamics in ST cells and IPEC-J2 cells. Collectively, the construction of the reverse genetic system may serve as a valid and effective tool for studying the function of proteins.

The PEDV spike protein is responsible for coronavirus adsorption and membrane fusion, mediating virus entry into host cells. Its core neutralizing epitope (COE) region is responsible for recognizing and binding to cell receptors, and inducing the host organism to produce neutralizing antibodies (Wang et al., 2017; Ji et al., 2021). The COE gene is highly conserved among different PEDV strains, therefore, vaccines based on the COE protein can provide cross-protection against G1 and G2 type variants (Lin et al., 2014). COE, as an antigen, can activate multidimensional immune responses including mucosal immunity, cellular immunity, and humoral immunity (Wang et al., 2019; Zheng et al., 2021; Yan et al., 2024). Therefore, COE is a key target when developing vaccines. In our study, to verify the function of the reverse genetic system, TGEV TH-98 strain ORF3 was used as the insertion site for expressing foreign genes, PEDV SJH COE protein was used as the immunogenic protein, and the recombinant virus rTH-98-Δ3-COE-HA expressing the COE protein was rescued. The results suggested that the construction of the TGEV reverse genetic system can serve as an informative tool for studying the function of foreign proteins, and provide the theoretical basis for studying the immunogenicity of TGEV and its foreign proteins.

In recent years, research has shown that a recombinant coronavirus can induce a humoral immune response in the host. Cruz et al. rescued a recombinant virus expressing the PRRSV M protein and successfully induced a humoral immune response (Cruz et al., 2010). Ribes et al. evaluated the mouse model of recombinant viruses expressing rotavirus VP7, improving the immunogenicity of recombinant coronaviruses expressing foreign protein (Ribes et al., 2011). The above results showed that rTH-98-Δ3-COE-HA and rTH-98 successfully induced specific immune responses in mice. Furthermore, the COE-HA protein was successfully expressed in the form of non-structural proteins, indicating that TGEV ORF3 is a potential ideal insertion site that can carry foreign genes and express target proteins. However, the level of anti-PEDV COE protein-specific IgG is lower than that of anti-TGEV N protein-specific IgG in our study. We believe that the reason for this phenomenon may be that rTH-98-Δ3-COE-HA is copied *in vitro* and the COE-HA gene uses the transcriptional regulatory element of the ORF3 expression cassette, making its expression abundance lower than that of the TGEV N protein. In this study, the exogenous COE protein expressed by the recombinant virus induced neutralizing antibodies in mice, revealing the feasibility of using a coronavirus as an antigen vector to construct recombinant virus vaccines.

Although the accessory proteins of coronaviruses are not essential factors for virus replication, they typically play a critical role in the infection and pathogenesis of the virus. The ORF8 of SARS-CoV-2 rapidly evolved to enhance its immunosuppressive function, promoting human to human transmission (Hurtado-Tamayo et al., 2023). Cruz et al. found that TGEV ORF7 antagonizes host antiviral response by binding to cell phosphatase PP1, reducing the phosphorylation of eIF2α and RNase L activation (Cruz et al., 2011). Moreover, after the deletion of ORF7, TGEV produced clinical symptoms earlier and more pronounced in piglets compared to the parental strain. The fundamental reason for the increased virulence of the recombinant virus is due to its intensified pro-inflammatory response in piglets, indicating that the deletion of ORF7 enhances the pathogenicity of the recombinant virus to piglets (Cruz et al., 2013). Porcine coronaviruses can also induce a significant increase in the expression of inflammatory cytokines during viral infection, such as TGEV (Ding et al., 2017), PEDV (Jung et al., 2020), and PDCoV (Jung et al., 2018). In Guo’s study, TGEV ORF3b suppressed Bcl-10 expression, activation of the NF-κB pathway, and TNF-α production by uniquely upregulating miR-885-3p expression (Guo et al., 2021). In PEDV, ORF3 interacts with IκB kinase β to promote NF-κB promoter activity mediated by IκB kinase β, and it downregulates the activation of IKBKB-meditated IFN-β promoter and expression of IFN-β mRNA (Kaewborisuth et al., 2020). However, Wu et al. found that PEDV ORF3 blocks the phosphorylation and nuclear translocation of p65, and downregulates the expression of IL-6 and IL-8 (Wu et al., 2020). In this study, the expression of inflammatory cytokines was increased in IPEC-J2 cells infected with rTH-98 and TH-98. However, there was no significant difference between rTH-98 and TH-98. Therefore, IPEC-J2 cells were selected in this study because they were isolated from porcine intestine and may be more representative of the physiology of porcine infection with the virus (Baliji et al., 2009; Kaewborisuth et al., 2018). However, the expression levels of inflammatory cytokines in IPEC-J2 cells infected with the recombinant virus rTH-98-Δ3-COE-HA were significantly lower than those in IPEC-J2 cells infected with rTH-98 and TH-98. The above results exhibited that TGEV infection can lead to the secretion of inflammatory cytokines in IPEC-J2 cells, suggesting that ORF3 enhances the inflammatory response after TGEV infection, and ORF3 may play a key role in inducing host cell immune responses.

In conclusion, we successfully established the reverse genetic system for the TGEV strain TH-98 and rescued the recombinant virus rTH-98-Δ3-COE-HA. In addition, the results prove that ORF3 is a non-structural protein that promotes TGEV replication. Our study illustrates the regulation of ORF3 in the host cell signaling pathway and the construction of recombinant vaccine virus, and provides a theoretical basis for revealing the role of ORF3 in virus replication and pathogenesis.

## Data Availability

The original contributions presented in the study are included in the article/**Supplementary Material**. Further inquiries can be directed to the corresponding authors.

## Supplementary material

Supplementary Figure 1 mRNA expression levels of COE-HA and N in the infected rescue virus IPEC-J2 cells. Values are the mean ± SD of three independent tests. *** represents p <0.001 between the COE-HA group and N group.
