# Supplementary figures and images for: Establishment of an infectious clone of the porcine transmissible gastroenteritis virus and a study on the location and function of accessory protein 3
Source: Front Cell Infect Microbiol. 2025 Jun 11;15:1609022. doi: 10.3389/fcimb.2025.1609022 (PMC12187753; doi:10.3389/fcimb.2025.1609022)

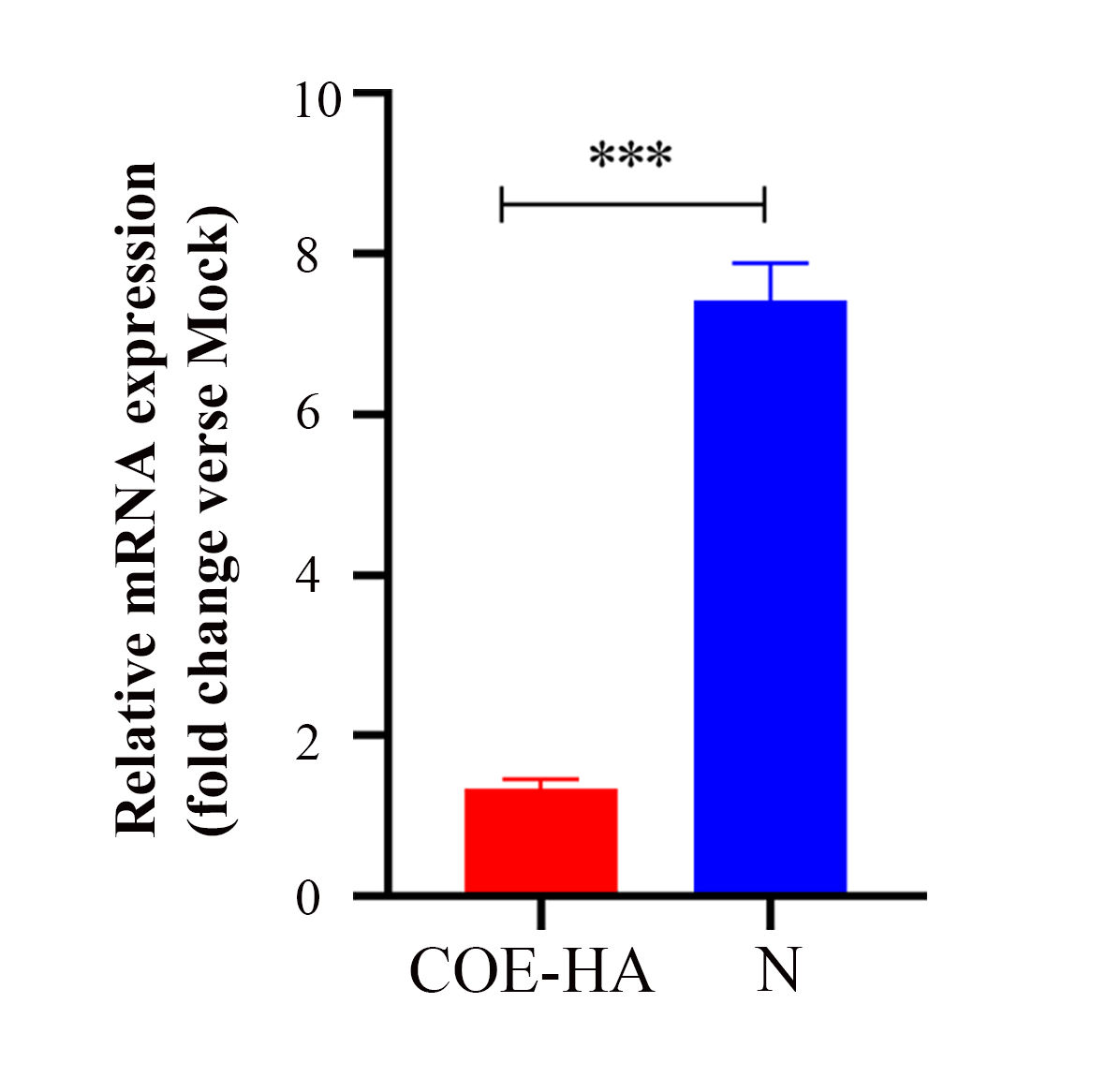

Supplement: Supplementary Figure 1 — mRNA expression levels of COE-HA and N in the infected rescue virus IPEC-J2 cells. Values are the mean ± SD of three independent tests. *** represents p <0.001 between the COE-HA group and N group. [file SupplementaryFile1.zip › Supplementary Figure 1.tif]
